# Indole Antitumor Agents in Nanotechnology Formulations: An Overview

**DOI:** 10.3390/pharmaceutics15071815

**Published:** 2023-06-25

**Authors:** Eleonora Russo, Carola Grondona, Chiara Brullo, Andrea Spallarossa, Carla Villa, Bruno Tasso

**Affiliations:** Section of Medicinal and Cosmetic Chemistry, Department of Pharmacy, University of Genova, Viale Benedetto XV, 3, 16132 Genova, Italy; carola.grondona@edu.unige.it (C.G.); chiara.brullo@unige.it (C.B.); andrea.spallarossa@unige.it (A.S.); carla.villa@unige.it (C.V.); bruno.tasso@unige.it (B.T.)

**Keywords:** indole, anticancer compounds, nanoparticles, drug delivery

## Abstract

The indole heterocycle represents one of the most important scaffolds in medicinal chemistry and is shared among a number of drugs clinically used in different therapeutic areas. Due to its varied biological activities, high unique chemical properties and significant pharmacological behaviors, indole derivatives have drawn considerable interest in the last decade as antitumor agents active against different types of cancers. The research of novel antiproliferative drugs endowed with enhanced efficacy and reduced toxicity led to the approval by U.S. Food and Drug Administration of the indole-based anticancer agents Sunitinib, Nintedanib, Osimertinib, Panobinostat, Alectinib and Anlotinib. Additionally, new drug delivery systems have been developed to protect the active principle from degradation and to direct the drug to the specific site for clinical use, thus reducing its toxicity. In the present work is an updated review of the recently approved indole-based anti-cancer agents and the nanotechnology systems developed for their delivery.

## 1. Introduction

Indole derivatives drugs can be of synthetic or natural origin; they have been studied as anticancer agents and used in clinical evaluation for their manifold pharmacological properties [1].

Among naturally occurring indoles with anti-tumor activity, vinblastine and vincristine, two tubulin inhibitors derived from Vinca rosea (also known as Catharanthus roseus), represent a main topic being used for the therapy of many types of cancer. Moreover, several synthetic indoles have been studied in recent years, some of which have entered clinical trials and been subsequently approved [2].

**Figure 1:**
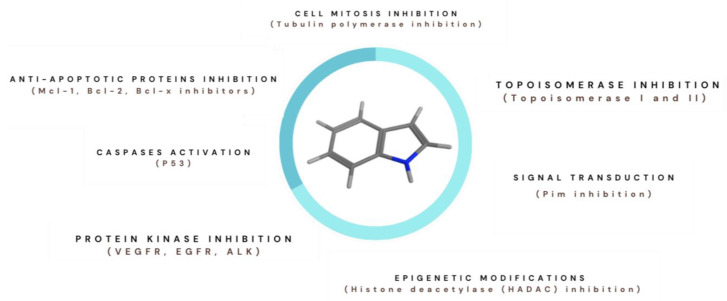
Schematic representation of mechanisms of action for indole derivatives.

All compounds present an indole core but also possess structural differences that justify different action mechanisms such as epigenetic modifications (HDAC inhibitors and SIRT inhibitors) acting on apoptosis (Mcl-1 inhibitors), cell signal transduction (Pim inhibitors) and cell mitosis (tubulin inhibitors). The most common actions are shown in Figure 1.

The wide structural variety of indole derivatives also causes dramatic variations in physical–chemical properties such as poor solubility and low bioavailability, while also presenting serious adverse side effects and multidrug resistance in patients. In order to overcome all these drawbacks, nanomedicine can be helpful in terms of conferring several advantages including an increase in drug stability, improvement in drug efficiency, and target site delivery at high concentrations together with controlled and sustained drug release [3,4].

In the present review article, we will discuss several indole-based anticancer agents approved by the U.S. Food and Drug Administration (FDA) for clinical use and applied in nanotechnology research for drug delivery.

### 1.1. Indole Derivatives and Cancer

**Figure 2:**
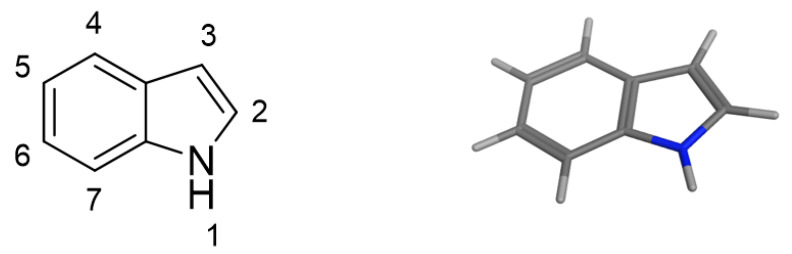
Three-dimensional structure of indole.

*1H-benzo[b]pyrrole* or indole (Figure 2) is an aromatic, highly rigid and planar heterocycle, initially isolated via treatment of the indigo dye, obtained from *Indigofera tinctoria*, with oleum. From a chemical point of view, indole structure can be easily derivatized in seven positions. Due to the delocalization of p-electrons, it easily undergoes electrophilic substitution and contains a slightly acidic NH group that can be converted into a nucleophilic reactive center under basic conditions [5,6]. In addition, the presence of a H bond-donating group ameliorates the binding to various biological targets [7].

The indole core is shared among a wide variety of natural products with diverse structural complexity [8], including the dimeric monoterpene indole alkaloids vincristine and vinblastine approved as chemotherapeutics agents for the treatment of several types of cancers (e.g., Hodgkin’s disease, Kaposi’s sarcoma, non-Hodgkin’s lymphoma, and neuroblastoma) [9,10,11,12,13]. The anticancer activity of indole derivatives is mediated by a wide variety of mechanisms such as apoptosis induction [14], tubulin inhibition [15], tyrosine kinase inhibition [16] and histone deacetylase inhibition [17], thus confirming the ability of the indole scaffold to interact with different pharmacological targets.

**Figure 3:**
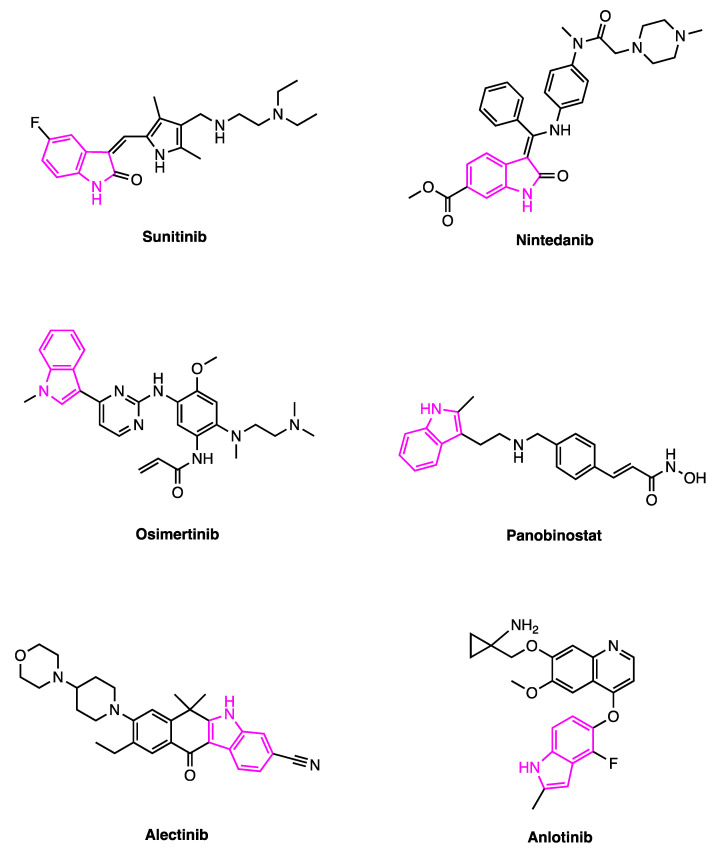
Chemical structure of selected synthetic indole derivatives approved for cancer treatment. Indole substructure is colored in pink.

For all these reasons, indole is considered one of the most important nitrogen-containing pharmacophores and it is designated as a “privileged scaffold” in medicinal chemistry for the identification of novel drugs active in different therapeutic areas including cancer. In this regard, in the last 10 years indole derivatives Sunitinib (SUNI), Nintedanib (NINTE), Panobinostat (PANO), Osimertinib (OSI), Alectinib (ALE) and Anlotinib (ANLO) (Figure 3) have been approved as novel antitumor drugs (Table 1) and further increase the therapeutic options against cancer, a leading cause of death worldwide, accounting for nearly 10 million deaths in 2020 [18].

Panobinostat (Farydak^®^, Figure 3) is an indole hydroxamic acid derivative developed by Novartis as a nonselective histone deacetylase (HDAC) inhibitor developed by Novartis. HDAC enzymes, along with histone acetylase enzymes (HAT), control the histones acetylation level of histones, playing a pivotal role in chromatin remodeling, gene transcription and expression regulation [20].The removal of acetyl groups from histones by HDACs prevents the transcription of genes encoding proteins involved in cell cycle regulation, cell differentiation, and apoptosis [20]. Histone acetylation deregulation and increased levels of HDAC are associated with the silencing of tumor suppressor genes and are typical alterations occurring in human cancer development [20,21]. In fact, increased HDAC levels have been reported in various solid human tumors, as well as hematological cancers and cancer cell lines, including multiple myeloma [20,22]. PANO was approved by the Food and Drug Administration (FDA) in 2015 in combination with bortezomib and dexamethasone for the treatment of patients with multiple myeloma who have received at least two prior treatments [23]. Moreover, in 2022 Panobinostat was designated as orphan drug for the treatment of glial tumors by the European Medicines Agency (EMA) [24]. To date, PANO as a single agent or in combination with other drugs is studied in ongoing phase II and III clinical trials for multiple myeloma treatment (NCT02654990 and NCT02506959), whereas other ongoing phase I clinical trials are principally focused on the treatment of glioma (NCT02717455, NCT04804709 and NCT04341311) [25,26].

Other indole-based derivatives such as Sunitinib, Nintedanib, Alectinib, Anlotinib and Osimertinib (Figure 3) are inhibitors of intracellular (TKIs) and receptor tyrosine kinases (RTKIs) are involved.

Intracellular and receptor tyrosine kinases play a pivotal role in signal transduction pathways and cancer development. Moreover, they are highly activated in malignant tumor cells but not in normal cells [27] and this make them a great target for cancer therapy. Activated forms of tyrosine kinase receptors can cause increases in tumor cell proliferation, induce antiapoptotic effects, and promote tumor growth, angiogenesis, and metastasis formation [28]. Although TKIs have a minor toxicity with respect to cytotoxic chemotherapy, they can lead to significant side effects including fatigue, diarrhea, hypertension and vomiting [28] that may cause the suspension of the therapy. Furthermore, the insurgence of drug resistance with various mechanisms is common in patients treated with TKIs [29,30].

For those reasons, many efforts have been made to develop new TKIs and this has led to a great number of clinical trials and approvals.

Sunitinib (Sutent^®^, Figure 3) is an indolinone, multi-target, anti-angiogenic first-generation TKI developed by Pfizer. Its targets are several members of the split-kinase domain family of RTKs, including vascular endothelial growth factor receptors (VEGFRs) types 1 and 2 (FLT1 and FLK1/KDR), the platelet-derived growth factor receptors (PDGFR α and β) and the stem cell factor receptor c-KIT, as well as the Fms-like tyrosine kinase 3 (FLT3) and RET kinases [31]. Although the relative contribution of each RTKs is still poorly understood, Sunitinib’s antitumor activity is mainly related to the PDGFR and VEGFR inhibition that reduces tumor vascularization, triggers cancer cell apoptosis, and causes tumor size reduction [31,32]. For this reason, this drug is approved as a first-line therapy for the treatment of advanced or metastatic renal cell carcinoma (RCC) and it is also used as a comparator drug in phase III clinical trials for the treatment of renal cell carcinoma [33]. Unfortunately, after a median of 6–15 months of treatment, cancer progression may occur in patients with RCC, indicating the occurrence of acquired drug resistance to Sunitinib [34] and underlining the need for second- and third-generation TKIs to overcome this problem. Sunitinib represents a first-line treatment for children for most common solid tumors such as neuroblastoma, central nervous system tumors, sarcomas and Wilms’ tumors [35,36].

Nintedanib (Ofev^®^, Figure 3) is an indolinone-derived intracellular tyrosine kinase inhibitor developed by Boehringer Ingelheim. NINTE simultaneously inhibits multiple kinases including fibroblast growth factor receptor (FGFR) 1, 2 and 3, VEGFR 1, 2 and 3 and PDGFR α and β. These receptors play an important role not only in angiogenesis but also in tumor growth and metastasis [37]. For this reason, NINTE was initially developed as anti-angiogenic agent. However, as the targeted kinases are also involved in lung fibrosis, it also provides clinical benefit in patients with idiopathic pulmonary fibrosis (IPF) [38], thus being approved by the FDA in 2011 as an orphan drug for the treatment of this condition [39]. In 2014, the FDA granted global approval of NINTE for the same indication [39]. Although current clinical trials are mainly focused on the treatment of lung diseases, there are also phase I and II clinical trials for the treatment of different types of cancers, including non-squamous (NsqNSCLC) non-small-cell lung cancer (NCT02299141 and NCT03377023) and advanced pancreatic cancer (NCT02902484).

Osimertinib (Tagrisso^®^, Figure 3) is a third-generation epidermal growth factor receptor tyrosine kinase inhibitor (EGFR TKI) developed by AstraZeneca for the treatment of advanced non-small-cell lung cancer (NSCLC). It is characterized by an indole nucleus directly linked to a substituted aminopyrimidine ring. Osimertinib targets various receptor variants (e.g., EGFR T790M) [40,41] selected in NSCLC patients by other EGFR TKIs [40]. In 2014, Osimertinib was granted orphan drug FDA designation for the treatment of EGFR mutation-positive NSCLC [42], and then approved by the FDA for the same indication in 2015 [42]. Phase II and III clinical trials are currently focused on its use, alone or in combination, for the treatment of NSCLC at different stages and characterized by different mutations (NCT03433469, NCT04035486 and NCT05020769) [43,44].

Alectinib (Alecensa^®^, Figure 3) is a highly selective second-generation anaplastic lymphoma kinase (ALK) inhibitor, developed by the Japanese company Chugai, a subsidiary of Roche [45]. From a chemical point of view, the compound differs from other available ALK inhibitors as it bears the rigid dihydro-5*H*-benzo[β]carbazole-3-carbonitrile scaffold. The ALK gene was initially identified as a novel fusion gene resulting from a chromosomal translocation in anaplastic large-cell lymphoma (ALCL) [46]. ALCL belongs to the group of high-grade non-Hodgkin’s lymphomas (NHLs). However, increased expression of ALK and ALK rearrangements have also been identified in solid tumors, including NSCLC [47].

ALE has a unique chemical structure, different from the structure of the scaffold of other available ALK inhibitors and, thus, it may overcome resistance to other ALK inhibitors caused by mutations [48]. Despite this, results of an in vitro study suggest that resistance to ALE may develop via the activation of bypass signals triggered by these receptor ligands [49]. In 2015, Alectinib was approved as an orphan drug for the treatment of patients with ALK-positive metastatic NSCLC who have progressed or are intolerant to Crizotinib, another TKI that was recently approved [50]. Subsequently, ALE was approved by the FDA and EMA in 2017 for the same indication. The currently ongoing clinical trials of this compound are mainly focused on NSCLC but also include studies on other solid tumors (NCT02091141 and NCT04632992).

Anlotinib (Focus V^®^, Chia Tai Tianqing Pharmaceutical Group Co.; Lianyungang, China, Figure 3) is simultaneously active on multiple kinases including FGFR 1, 2 and 3, VEGFR 1, 2 and 3 and PDGFR α and β [51,52]. It has a broad spectrum of inhibitory effects on angiogenesis and tumor growth [53]; in particular, it inhibits cell migration and possesses stronger anti-angiogenesis activity than do other anti-angiogenic agents such as SUNI and NINTE [51]. Structurally, this compound is characterized by an indole moiety linked via an ether functionality to a quinoline ring. In 2018, ANLO was approved by the China National Medical Products Administration (NMPA) for the treatment of patients with locally advanced or metastatic NSCLC that have undergone progression or recurrence after more than two lines of systemic chemotherapy [54]. In the same year, ANLO was approved in Europe by the EMA as an orphan drug for the treatment of soft tissue sarcoma [55]. To date, this RTKI, alone or in combination, is currently studied in phase II and III clinical trials for the treatment of different cancer types, including thyroid cancer (NCT05007093), and hepatocellular carcinoma (NCT04213118 and NCT04172571) [56,57,58].

### 1.2. Nanoparticles for Cancer Treatment

Thanks to the rapid development of nanotechnology and nanoscience, a great variety of particles of different sizes, shapes, and structures are now available for anticancer therapy. The efficacy of conventional chemotherapy based on low-molecular-weight drugs (MW < 1000 Da) is limited by the pharmacokinetics and biodistribution of the drug’s storage in healthy organs and tissues. To overcome these limitations, nanotechnology proved to be an excellent strategy as it reduced renal clearance and increased drug accumulation at the target site [59]. Small drug molecules are distributed in the full body, resulting in a very large distribution volume (Vd) and requiring high doses to reach the target site, often leading to toxic side effects [60]. Nanocarriers become entrapped in tumor cells or accumulate around them, releasing the drug safely and specifically to the mutated cells, increasing the drug’s bioavailability and minimizing its exposure to the healthy tissues [61]. In addition, due to their specific biocompatibility, low toxicity, and low immunogenicity, nanoparticles (NPs) are an efficient delivery system and in recent years, various types of nanoparticulate delivery systems have been investigated as potential drug carriers. A great variety of NPs are available that can be divided into two categories: (a) inorganic NPs (e.g., fullerene, quantum dot, abd metallic nanoparticles); (b) organic NPs (e.g., polymeric, protein based, micelle, liposome, solid lipid nanoparticles, dendrimer, nanotube and nanofibers, nanogels and scaffold matrices) [62].

In recent years, several new nanoformulations have been investigated as effective drug delivery systems for indole-containing compounds SUNI, NINTE, PANO, OSI, ALE and ANLO. In the current review, the developed formulations will be discussed and critically analyzed to highlight the advantages and the limitations of the set of nanodelivery systems.

## 2. Sunitinib

Sunitinib (Sutent^®^, Pfizer) is commercially available as an oral immediate-release hard capsule (dosage: 12.5, 25, 37.5 and 50 mg) formulated with Mannitol (E421) (diluent), Croscarmellose sodium (binder and disintegrant), Povidone (K-25) (solubilizing agent and disintegrant), Magnesium stearate (lubricant) and Gelatin as inactive ingredients [63]. Other oral pharmaceutical forms, such as suspension, have also been investigated for pediatric therapy [64]. Recently, the research has turned toward more innovative and advantageous drug delivery compared to conventional pharmaceutical forms, and some of these studies are reported as examples of Sunitinib delivery using nanotechnology.

### 2.1. Liposome–Sunitinib

One of the first studies on liposome-SUNI application was focused on lipoplexes (cationic liposome/DNA complexes) prepared with 1-palmitoyl-2-oleoyl-sn-glycero-3-ethylphosphocholine (EPOPC) and cholesterol (Chol). Cationic liposomes in a 4/1 lipid/DNA (AMOs) (+/−) charge ratio, associated with human serum albumin (HSA) and containing combinations of SUNI and different microRNAs, were prepared and tested against pancreatic ductal adenocarcinoma (PDAC) [65]. The HSA-EPOPC:Chol/AMOs (+/−) (4/1) formulation reveals a neutral zeta potential of 0.4 ± 1.5 mV and a mean diameter of approximately 450 nm. Hs766T cells (PDAC cell lines) were transfected with lipoplexes, showing, under confocal microscopy, internalization into tumor target cells, and delivered their content into the cell cytoplasm. This nanosystem induces an inhibition of microRNAs (miR-21, miR-10b, miR-221, and miR-222) that are unusually expressed in the cancer model presented in the paper.

Hu et al. [66] studied liposome-carrying microbubbles containing SUNI, released with or without ultrasound (US), to reduce drug adverse events in the treatment of renal cell carcinoma (RCC). The liposome was made of hydrogenated soy phosphatidylcholine and cholesterol, while the microbubbles were prepared by mixing nonionic surfactant Span-60, Tween-80, and polyethylene glycol (PEG). The mean diameter of the microbubbles was 3.17 μm, while the liposomes size was 165 nm and the SUNI entrapment efficiency in the liposomes was approximately 78%. In this study, GRC-1 cell lines were used for in vitro studies, whereas nude mouse cancer models were used for in vivo evaluations. The obtained results indicated a remarkable inhibition of the human GRC-1 cells in vitro, decreasing cell survival rates and increasing apoptosis rates. In addition, in vivo experiments showed that tumor growth treated with the sunitinib-loaded microbubble + US mouse group increased slowly over the same period in respect to that of other groups.

Another paper [67] reported a dual-function drug delivery system comprising the near-infrared dye IR-780 as a photothermal agent and SUNI incorporated into liposomes (Lip-IR 780-Sunitinib). The encapsulation efficiency (EE%) for IR 780 and SUNI was 66.59% ± 0.02 and 90.12% ± 0.31, respectively. The particle size of Lip-IR780-Sunitinib was approximately 150 nm, measured using transmission electron microscopes (TEM) and dynamic light scattering. The liposomal formulation stability was good at a pH of 5.0, 6.8 or 7.4 during the 24 h incubation period. The synergistic photothermal anti-tumor and anti-angiogenic effects of this nanosystem were studied in vitro and in vivo, showing potential for clinical use in tumor therapy.

Jao et al. [68] also exploited near-infrared (NIR) light stimulation for SUNI release to obtain antiangiogenic and antitumor effects. The authors reported a smart porphyrin-based nano-delivery system—iRGD peptide-modified Pp18-lipos carrying SUNI (called iPlipo-SUN). Four groups of liposome-like nanoporphyrin, Pp18-lipo (Plipo), iRGD-modified Plipo (iPlipo), SUNI-loaded Plipo (Plipo-SUN), and SUNI-loaded iPlipo (iPlipo-SUN), with diameters in a reasonable range (around 100 nm), were designed. iPlipo-SUN morphology demonstrated a nearly spherical shape with favorable monodispersity. The zeta potential of Plipo, iPlipo, Plipo-SUN, and iPlipo-SUN was separated and the values were −33.48 ± 1.45 mV, −39.95 ± 1.06 mV, −40.49 ± 1.43 mV, and −40.99 ± 2.40 mV, respectively. In vitro experiments (on the cytotoxicity of liposomes) and in vivo experiments (on biodistribution including evaluation of the antiangiogenic effect, antitumor effects and side effects after treatment in mice) were also carried out. The obtained results confirmed that iPlipo-SUN could achieve preferably intratumoral enrichment and superior anticancer effectiveness, in addition to achieving angiogenesis inhibition.

In the last two and more recent papers, SUNI was incorporated into liposomes for the treatment of solid tumors such as glioblastoma [69] and melanoma [70].

In the glioblastoma brain tumor, matrix metalloproteinase-2 (MMP-2) and chloride channel-3 (CIC-3) are up-regulated, causing glioma progression and invasion. For this reason, the liposomes containing SUNI were functionalized with chlorotoxin (CTX), a scorpion venom-derived peptide with a high affinity for MMP-2 and ClC-3. The average size of all liposomes studied was in the range of 138–149 nm with a narrow distribution of the polydispersity index (PDI < 0.3); the zeta potential of liposomes with CTX-SUNI was −2.47 ± 0.13 mV and that for SUNI alone was −1.78 ± 0.12 mV. The EE% of SUNI for both liposomes was more than 96% and the stability was good after 90 days under the storage condition. Various tests were carried to verify liposome cytotoxicity, cellular uptake and the internalization mechanism. The authors concluded that the functionalized liposomes showed specific cellular uptake by glioblastoma cells, inactivate MMP-2 complexes and can be used as an effective SUNI nanocarrier for the modulation of several different pathways involved in glioblastoma tumorigenesis, including neo-angiogenesis, migration, proliferation, apoptosis and autophagy.

Myeloid-derived suppressor cells (MDSCs) play an important role in the immune escape of various tumors such as melanoma and for these reasons they are considered an important target in tumor immunotherapy. SUNI can directly deplete MDSCs through the inhibition of STAT3 phosphorylation and thereby reverse the immunosuppressive tumor environment.

Lai et al. [70] showed the efficacy of liposomes containing Doxorubicin and SUNI (DS@HLipo) in extending drug circulation in the blood and in improving accumulation, penetration and release in tumor sites. The physicochemical characteristics of DS@HLipo were investigated; in particular, the particle size was in the range of 150–180 nm, the zeta potential was around −10 mV and the stability was studied in phosphate-buffered saline (PBS) with 10% fetal bovine serum (FBS) at 37 °C for at least 48 h, suggesting its good stability in circulation. Furthermore, the liposomes also maintained colloidal stability in PBS at 4 °C for 7 days. In addition, light irradiation proved to increase the release rate of SUNI (100% in 5 min with light; 20% in 24 h without light) in in vitro tests. In vivo anticancer activity was explored in the B16F10 tumor-bearing C57BL/6 mouse model and the results revealed that the treatment, not only more effectively decreased the immunosuppressive cells (MDSCs, Treg cells) and anti-inflammatory cytokines (TGF-β and IL-10), but also elevated the levels of antitumor cytotoxic CD8+ T cells and pro-inflammatory cytokine IFN-γ in tumors. This behavior demonstrated immune enhancement in melanoma therapy.

### 2.2. Chitosan Nanoparticles—Sunitinib

Chitosan NPs have always proven to be a very useful delivery system for both hydrophilic and hydrophobic drugs. They are able to control the release of the drug in a prolonged and sustained way over time allowing a homogeneous distribution at the target site and reducing side effects [71]. As a small molecule, SUNI can be encapsulated in this type of nanocarrier as reported in recent studies.

Joseph et al. [72] prepared SUNI-containing chitosan NPs whose physicochemical properties were characterized via scanning electron microscopy (SEM), Fourier transform infrared spectroscopy (FTIR), and powdered X-ray diffraction (XRD) techniques. The NP size was from 65 to 98 nm for the SUNI-unloaded and -loaded formulation, confirmed also via FTIR. The EE% was in the 97% to 98% range depending on the concentration of the drug in the nanocarrier. The release profile showed an initial burst effect of around 20%, followed by a slow and increased release rate of 70% of SUNI within 72 h. These results indicated that this nanocarrier was a good candidate for SUNI delivery.

In a second paper, Saber et al. [73] reported the preparation and characterization of cyclic peptide cRGD-(Chitosan-SUNI-Au) NPs (cRGD (CS-STB-Au) NPs_ for targeting tumor vasculature and improving SUNI bioavailability, reducing drug dose administration, and increasing patient compliance. The average size of NPs was found to be about 50 nm, and they were highly stable and monodisperse. At 37 °C in PBS solution, about 50% of SUNI was released within the first 5 h and then, a sustained release profile was observed in the following 48 h. A cytotoxicity assay indicated that cRGD (CS-STB–Au) NPs significantly reduced cell proliferation compared to the free drug.

Recently, Jafari et al. reported the use of chitosan-based NPs containing a magnetic polymer [74,75]. Magnetic cellulose-derived mHPMC and mCMC were introduced in the preparation of chitosan core–shell nano-carriers (namely mHPMC@Chitosan and mCMC/CTS) encapsulating SUNI. The mHPMC@Chitosan NPs had a diameter of 123 nm at pH = 7.4 but swelled to about 290 nm at pH = 4.5. The EE% was around 90% and the cumulative release of SUNI was about 62% during a 6 h period and it reached 93% until 2 days at pH = 4.5. No release was observed at pH = 7.4. These results highlighted that the magnetic nanocarrier represents a valuable pH-responsive system for the sustained release of SUNI. The mCMC/CTS NPs, in addition to SUNI, contained saffron (SAF) as a bioactive compound, and demonstrated a spherical shape with a porous morphology and mean size of 35 ± 5 nm via SEM analysis. Additionally, in this case, the release of the two drugs was too slow at a neutral pH, while at an acidic pH a slightly rapid release of SUNI and SAF occurred, which reached 18% and 25% after 200 min, respectively. The total drug released was obtained after 3 days. Moreover, SUNI and SAF nanocarriers showed cytotoxic and antibacterial properties against MCF-7 cancer cells and *S. aureus* bacteria, respectively.

Chitosan-based NPs crosslinked with k-carrageenan with magnetic properties were also investigated by Karimi et al. [76]. In this study, magnetic carriers were generated through the in situ co-precipitation of iron ions in the presence of chitosan with different molecular weights and then adding κ-carrageenan solutions to achieve crosslinking. Magnetic properties, surface morphology, polymeric swelling, SUNI loading and release were analyzed. NPs showed superparamagnetic behavior; the surface of all magnetic carriers prepared contained quasi-spherical nanoparticles with a porous morphology, their diameters were approximately 45 nm and the degree of swelling increased as the molecular weight of chitosan increased. The EE% was around 70%, at all used pHs (7.4, 5.8 and 4.5); the SUNI release rate occurred fast in the first 90 min and the burst release of drug may be attributed to the ionically electrostatic interaction with k-carrageenan. Interestingly, SUNI release was completed for 7 days.

### 2.3. Magnetic Nanoparticles–Sunitinib

Magnetic NPs have proven to be very important tools in the treatment of cancer, especially when associated with hyperthermia treatment (HPT) that consists of the use of heat (in the range of 41–46 °C) to induce effective cellular shock damage [77]. In the last decade, metallic NPs have been largely exploited for SUNI delivery as exemplified below.

Chen et al. [78] presented supermagnetic iron oxide nanoparticles (SPIOs) coated with bovine serum albumin (BSA) containing a combination of Curcumin (Cur), a depressor of chemo-resistance, and SUNI to enhance the antitumor effect. The NPs were obtained via a modified co-precipitation method in the presence of BSA (which facilitated the in situ immobilization of crystallized SPIOs) and had a hydrodynamic diameter of 53.1 ± 4.3 nm, which further increased to 75.6 ± 4.6 nm after encapsulating drugs. The increase in the zeta potential value after drug loading (*ζ* = −34.6 mV before loading; *ζ* = −32.1 mV after loading) indicates the high colloidal stability of the BSA-SPIOs. The EE and drug loading (DL) capacity were 99.8 ± 3.2% and 7.0 ± 0.2% for SUNI, and 100 ± 0.1% and 13.1 ± 0.01% for Cur, respectively. In this nanocarrier, both drugs displayed sustained release behavior compared to that of free drugs, demonstrating that BSA-SPIOs would be excellent vehicles. Interestingly, drug release was similar both in acidic-pH (5.4) and neutral PBS. The antitumor effects of BSA-SPIOs co-loaded with SUNI and Cur were assessed in an MCF-7 xenograft mouse model; in vivo pharmacokinetic analysis demonstrated that BSA-SPIOs delivered the encapsulated drugs to the tumor site and, at the same time, maintained the efficient concentrations for therapeutic activity.

Zhang et al. [79] investigated folic acid (FA)-modified zirconium core-metal–organic-framework (MOF) Uio-66 as a metallic nanocarrier to deliver indocyanine green (ICG) and SUNI for hepatocellular carcinoma (HCC) combination therapy. The nanocarrier displayed a diameter of about 50 nm, and the drug loading and encapsulation efficiency of SUNI were 2.52 ± 0.31% and 75.67 ± 5.57%, while those for ICG were 3.05 ± 0.53% and 95.36 ± 3.25%, respectively. The drug release experiment was carried out in a cell culture medium; the release profile was fast in the first 6 h and became almost steady after 10 h. The cytotoxicity and biocompatibility of the fully encapsulated NPs were evaluated in HepG2 human HCC cells; the combination of the two drugs emerged to be more potent than single-drug loading. In addition, NPs expended an excellent cytotoxic effect against HepG2 cells suggesting a substantial impact in cancer treatment.

Recently, Torabi et al. [80] reported magnetic mesoporous silica nanoparticles (MMSNPs) as multifunctional drug delivery systems endowed with some unique characteristics: high loading capacity, high surface area, tunable pore diameters, and biocompatibility with a non-toxic nature. In particular, magnetic nanocarriers were loaded with MUC-1, a glycoprotein encoded by the mucin 1 gene overexpressed in the most malignant epithelial cell surface, and SUNI for ovarian cancer treatment. The MMSNPs’ sizes were about 97.6 nm with a zeta potential of +10 mV and narrow PDI of 0.1. The EE% of SUNI was 90 ± 0.36% and the release behavior was pH-dependent; the mean cumulative release indicated that the pH reduction from 7.4 to 5.4. increased SUNI release from about 10% to 60%, after 24 h. As assessed via flow cytometry analyses, the MMSNP-SUN-MUC-1 showed high internalization and uptake in OVCAR-3 cells that died by apoptosis.

### 2.4. Solid Lipid Nanoparticles–Sunitinib

To overcome disadvantages presented by liposomes, some studies about solid lipid nanocarriers (SLNs) as delivery system for SUNI were recently reported in the literature.

A novel nanostructured lipid carrier (NLC) modified with biotin was prepared by Taymouri et al. [81] via the emulsion solvent diffusion and evaporation method and loaded with SUNI. The obtained biotin-SUNI-NLCs showed a mean size value in the 125.5–410.63 nm range, a zeta potential of around +10 mV, an EE% of between 67.28% and 92.02% and a release profile in the range 28.84–64.47%. Finally, the cellular uptake of biotin-SUNI-NLCs was higher that of the free drug and significantly reduced the proliferation of lung cancer A549 cells.

Khaledian et al. [82] suggested two natural lipids (namely, fat tail (FT) and Roghan Kermanshahi oil) to prepare SLNs for SUNI delivery. SLNs were fabricated using the modified solvent evaporation–ultrasonic combination method and then coated with chitosan and tragacanth gum to improve the efficacy in drug delivery and to increase the adsorption and mucoadhesive properties of nanocarriers. The SLNs were characterized for their size, zeta potential, PDI, morphology, and thermal properties of different long-chain fatty and polymer coatings. Furthermore, the release kinetics and the cytotoxicity of the prepared SLNs with and without drugs were evaluated. The results reported showed that the size of SLNs prepared with fat tail was smaller than samples obtained with Roghan Kermanshahi (86 nm vs. 118 nm), and coating with chitosan in both lipids did not affect the size of the particles (97 and 132 nm, respectively), but the addition of tragacanth led to a size of 110 and 156 nm for both systems, respectively. The zeta potential for uncoating SLNs was around −30 mV, while that for for chitosan-coated SLNs was +30 mV. The EE% and DL% of both formulations (i.e., polymer-coated FT-SLNs and polymer-coated Roghan SLNs) were 97% and 22%, and 95% and 27%, respectively. The release profile showed that after 96 h, more than 95% of SUNI was released from FT-SLNs while in the polymer-coated FT-SLNs the release percentage was 74%. In the case of Roghan-SLNs, after 96 h, more than 98% of SUNI release occurred, while for polymer-coated Roghan-SLNs it reached 88%. The formulations had no significant toxicity even at the highest concentrations. All SLNs loaded with SUNI showed greater inhibition of cell viability compared to that of the free drug in THP-1 cells. Collectively, these formulations can be a good lipid nanocarrier for SUNI delivery.

Recently, Ahmed et al. [83] proposed new lipid polymer hybrid NPs (LPHNPs) containing SUNI for breast cancer treatment. These NPs were prepared with lipoid-90H and chitosan, using lecithin as a stabilizer, via the emulsion solvent evaporation method. The optimized formulation showed the following properties: size = 439 ± 5.8 nm, PDI = 0.269, zeta potential = +34 ± 5.3 mV, and EE% = 83.03 ± 4.9%. In vitro SUNI release was found to be 84.11 ± 2.54% after 48 h, as compared to that of the free drug at 6 h which was 24.13 ± 2.67%. In an MTT assay on breast cancer MCF7 cells, the formulation exhibited anticancer activity, due to the enhancement of SUNI release. Furthermore, the ELISA assay highlighted an increase in caspase 3, 9 and p53 production, confirming apoptotic activity.

### 2.5. Micellar Nanocomplex–Sunitinib

The micellar nanocomplex (MNC) is a new technology in drug delivery system (DDS) for cancer therapy [84]. The MNC uses hydrophobic interaction (and not conjugation) to maintain complex drug molecules in NPs and a micellar system is obtained through special self-assembly polymers (e.g., PEG). Several papers in the literature exploited this new technology to deliver SUNI and some examples are given below.

Yongvongsoontorn et al. [85] proposed a SUNI-loaded micellar nanocomplex (SUNI-MNC) obtained via the self-assembly of SUNI and PEG-conjugated epigallocatechin-3-O-gallate (PEG-EGCG). Small-sized particles and the high drug loading capacity of SUNI-MNC were achieved at high hydration temperatures due to the formation of compact structures with strong SUNI−EGCG interactions. SUNI-MNC showed a neutral surface charge, demonstrating that PEG covered the SUNI core. The SUNI-MNC carrier showed enhanced anticancer effects and less toxicity than did orally/intravenously administered SUNI on human renal cell carcinoma-xenografted mice, demonstrating efficient effects on angiogenesis, proliferation and apoptosis. Compared to conjugated PEG-poly(lactic acid (PEG-PLA), the micellar system was found to have higher efficacy and lower toxicity.

Zeng et al. [86] synthesized the sialic acid-PEG-ibuprofen (SA-PEG-IBU) amphipathic conjugate which can self-assemble into nanomicelles and can be loaded with SUNI in aqueous solution (SPI/SUNI). The nanomicelles presented a spherical shape with a diameter of 21.42 ± 0.25 nm and a zeta potential value of −5 mV, measured in PBS-added 10% serum for up to 7 days. The drug loading and EE% were 8.3% and 91.3%, respectively. SUNI releasing rate gradually decreased within 8 h under various pH levels (7.4, 6.8 and 5.5), showing faster release at pH 6.8 and 5.5. In in vivo test, SPI/SUNI nanomicelles displayed ideal tumor targeting capacity as they accumulated in the tumor from 3 to 24 h. Furthermore, histopathology analysis revealed that the nanomicelles did not cause visible damage to the main organs, including the heart, lung, liver, spleen, and kidney.

In other research articles, SUNI was encapsulated in nanomicelles together with Paclitaxel (PTX) [87,88]. In the first work, He et al. [87] prepared the pH-responsive poly (aspartic acid-dibutyl-1,3-propanediamine) (PAsp(DBP)) micelle core (MC), in which PTX was loaded. In addition, SUNI was encapsulated into β-cyclodextrin and then conjugated with MC. The PTX-SUNI-MC had a hydrodynamic diameter of 183 ± 34.7 nm and a spherical morphology, and the drug loading was 5.34% for PTX and 1.12% for SUNI. The cumulative release of SUNI from nanomicelles reached 60% after 8 h in the presence of MMP-2. However, after the addition of an MMP-2 inhibitor, the release percentage decreased below 25% after 36 h as observed without MMP-2. The nanoformulation showed in vitro cell cytotoxicity, cell apoptosis and anti-angiogenesis activity. Moreover, PTX-SUNI-MC was able to effectively accumulate at tumor sites, to release SUNI into the tumor matrix and PTX inside tumor cells. In the second work by Qin et al. [88], polymeric micelles composed of poly (styrene-co-maleic anhydride) (SMA) were prepared via a self-assembly process and loaded with PTX and SUNI. The PTX-SUNI micelles showed diameters of 114.8 ± 2.96 nm with a PDI of <0.20, a negative zeta potential of −42.73 ± 0.12 mV, and drug loading and EE% for PTX and SUNI of 7.06 ± 0.01% and 15.89 ± 0.02%, and 27.63 ± 0.03% and 75.55 ± 0.02%, respectively. The release rate of SUNI from the micelles was 38.21% after 8 h and 91.03% after 48 h, while for PTX release it was only 20.06% within 8 h and 37.30% within 48 h. Collectively, the nanomicellar co-delivery of PTX and SUNI promoted the maturation of dendritic cells and immune responses, inducing cancer cell apoptosis, resulting in a strong form of synergistic chemo-immunotherapy for breast cancer.

Another similar research involved the combination of SUNI with the irinotecan derivative (SN-38) [89]. The authors prepared and characterized methoxy PEG-poly(ε-caprolactone) (mPEG-PCL) polymeric nanomicelles co-loaded with SN-38 and SUNI for colorectal cancer treatment. Depending on the drug/polymer ratio, the nanomicelle size varied from a minimum of 88.8 nm to a maximum of 112.8 nm, the PDI was 0.116, drug loading and EE% of SN-38 were 2.39% and 98.70%, respectively, and those of SUNI were 14.73% and 86.41%, respectively. Within 72 h, about 35% of the SN-38 and 85% of the SUNI had been released from the nanomicelles. The combined treatment of SN-38/SUNI micelles inhibited the viability of three colorectal cancer cell lines (namely, HT-29, SW-620, and HCT-116 cells) and accumulate in the tumor at a higher concentration than that with SUNI alone.

Braatz et al. [90] investigated micelles consisting of dendritic (poly glycerol sulfate)-SS-poly(ε-caprolactone/poly(lactide)/poly(lactide-co-glycolide)) indicated by the codes dPGS-SS-PCL, dPGS-SS-PLA, and dPGS-SS-PLGA containing SUNI. The nanomicelles were formed in PBS via nanoprecipitation with acetone and subsequent solvent evaporation and showed a hydrodynamic radius of about 100 nm, a surface charge of −44 mV, and drug loading and EE% of 13% and 65%, respectively. The stability of the formulation was about 94% after 24 h in the presence of an elevated serum protein concentration. The micelles showed low drug leaching with a value of 20% after 24 h under physiological conditions (pH 7.4), which was constant for the next 4 days. In the presence of GSH (pH 5) or GSH/Novozyme (pH 5) the drug release increased to 42% after 24 h and reached 70% (GSH) and 85% (GSH/Novozyme) after 5 days. Compared to the free drug, the nanomicelles led to a 10-fold enhancement of SUNI antitumor activity with no toxicity or cell suffering associated.

### 2.6. Miscellaneous Nanoformulations-Sunitinib

Other systems were reported in the literature for SUNI delivery against different solid tumors. These alternative nanoformulations include hydrogels [91,92,93,94,95], PLGA NPs [96], functionalized carbon nanotubes [97] and nanosomes [98]. Table 2 shows all the nanotechnological systems presented in this review for Sunitinib.

## 3. Nintedanib

As previously reported, NINTE was successfully developed by Boehringer Ingelheim and then approved by the FDA in 2014. The dose of the NINTE commercial soft capsule is 120 mg. It was used also to treat idiopathic pulmonary fibrosis diseases which can only be treated through lung transplantation. Therefore, due to its poor solubility (around 4.7%), new delivery systems were investigated that can load a higher amount of the drug and overcome the solubility problem.

For this type of indole, the innovative formulation most investigated in the literature was that of albumin nanoparticles [99,100,101].

Xu et al. [99] prepared albumin NPs via coaxial electrospray technology, that could fabricate nanofibers or micro/nanoparticles by conducting a high voltage from a metal capillary tip to induce a charge on the droplet’s surface. The obtained NPs contained Bufalin (BF), an anti-tumor steroid from Chinese medicine, and NINTE. The results highlighted that the nanocarrier exhibited a spherical morphology with good dispersion and a smooth appearance; the particle size detected via DLS was 879 ± 56 nm, the PDI was 0.16, and the zeta potential was −5.86 ± 1.12 mV. The EE% was 78.0% for BF and 76.2% for NINTE. This preparation method exhibited versatility for encapsulating many drugs, indicating that the albumin NPs could be used as a multi-drug platform. The in vitro release profiles of BF and NINTE were examined in a pH 7.4 PBS medium (0.1% Tween 80) with or without serum. The results demonstrated that BF and NINTE had a sustained release without a burst effect when analyzed in a nanocarrier in contrast to when it was analyzed as a free drug. In addition, the release of two drugs from the formulation was rather slow in the presence of serum, indicating the excellent stability of albumin NPs. The nanocarrier showed in vitro synergistic anti-tumor activity against HepG2 cells in a dose-dependent manner; in addition, NPs increased drug uptake in tumor cells through guanidine-based membrane affinity and ursolic acid (UA)-mediated active transport, thus increasing cancer cell apoptosis.

Zha et al. [100] proposed two types of NINTE-albumin NPs identified as ND-FSA NPs (containing folate) and ND-BSA-NPs (without folate). The characterization of both formulations showed an average particle size for ND-BSA NPs of 316.47 ± 30.25 nm and for ND-FSA NPs of 764.68 ± 88.46 nm, with a spherical shape and smooth outer surface. The two kinds of formulations had a negative potential (*ζ* = −31.39 ± 1.45 mV and *ζ* = −18.38 ± 0.41 mV, respectively). The DL of NINTE in NPs was 3.92 ± 0.19%, while the EE% was 87.46 ± 0.66%. In PBS, after 24 h the release of NINTE from the nanocarriers proved to be higher than that of the free drug (64.49% vs. 43.29%), thus indicating the excellent water solubility of albumin and folate which improved the NINTE release rate. Cytotoxicity and cell uptake experiments indicated an enhanced uptake ability and a significant inhibition of breast cancer cell MCF-7.

Xu et al. [101] also considered BSA NPs decorated with the biguanide group (p-biguanidinobenzoic acid, CBH) containing NINTE (NINTE-CBH-BSA NPs). The biguanide group was introduced for its great penetrating ability into the membrane lipid bilayer and for its potential antitumor activity. NINTE-CBH-BSA NPs possessed a regular morphological shape and uniform particle size with an average diameter of 47.2 ± 0.44 nm, and a zeta potential value of −25.4 ± 1.40 mV. The EE% and DL% were about 79.3 ± 0.46% and 16.5 ± 0.07%, respectively. The nanocarrier presented good storage stability for 48 h. The release profile of NINTE-CBH-BSA NPs in pH 7.4 PBS showed evident differences between the NINTE free drug and that encapsulated in NPS; free NINTE was released to about 70% at 6 h vs. 24 h for the encapsulated form, and 90% was reached after 12 h for the free drug while less than 50% was reached until 12 h for NINTE in NPs. Furthermore, this nanoformulation exhibited inhibitory effects on cell proliferation, the biguanide group increased cell uptake and the NINTE-CBH-BSANPs enhanced the anti-tumor effect of the drug.

To improve oral bioavailability, other nanocarriers were studied for NINTE delivery including liposomes [102,103] and solid lipid nanoparticles [104].

Kala et al. [102] developed D-alpha-tocopheryl polyethylene glycol 1000 succinate (TPGS) liposomes to increase the oral bioavailability and dissolution efficiency of the marketed NINTE formulation. The optimized formulation, studied using an experimental design, was evaluated in terms of size (121 ± 4 nm), PDI (0.214 ± 0.041), zeta potential (+45 ± 2.6 mV) and EE% (87.5 ± 4.4%). The liposomes were freeze-dried using sugars to form a glassy amorphous matrix around the particle; the stability was good at different pH values (1.2 and 6.8), for up to 3 months under 25 °C/60% RH and 2–8 °C. The release profile showed prolonged behavior with 9% of the drug released in 2 h and 92% in 36 h following Higuchi kinetics. The liposomes demonstrated efficient internalization and cellular uptake in A-549 cancer cells and the higher bioavailability of NINTE, which was about 6.23-fold.

Kallus et al. [103] tested the encapsulation of NINTE into liposomes or into PLA NPs. As liposomal building blocks, 1,2-distearoyl-sn-glycero-3-phosphocholine (DSPC), cholesterol (CHOL) and 1,2-distearoyl-sn-glycero-3-phosphoethanolamine-N-[methoxy(polyethylene glycol)-2000 (DSPE-mPEG-2000) in a ratio of 55:40:5 mol/mol were used. The PLA NPs were synthesized using the nanoprecipitation method and showed particle sizes between 96 and 147 nm with a PDI between 0.09 and 0.14, and a very low EE% of 2–6%. The liposomes showed an average size of 98 ± 4 nm (PDI: 0.18 ± 0.04) and zeta potential of −2.1 ± 1 mV with an EE% value of 34%. Additionally, stability and release kinetics were investigated. The NINTE release profile revealed that after 48 h only 16 ± 3.9% of drug had been released, indicating very stable drug entrapment. The formulations were tested for their anticancer activity on lung cancer cell lines and their uptake kinetics were investigated using flow cytometry measurements. The promising results evidenced reduced toxicity and increased antitumor efficacy.

Patel et al. [104] described nanostructured lipid carriers (NLCs) made up of solid lipids (GMS) and Capmul MCM (mixture of medium-chain mono and diglycerides) prepared via high-speed homogenization followed by the probe sonication technique. The particle size of NINTE-NLCs was 125.7 ± 5.5 nm, the PDI value was 0.157 ± 0.031 and the zeta potential was −17.3 ± 3.5 mV. The EE% of the carrier was found to be 88.5 ± 2.5%, due to the high solubility of NINTE in GMS and Capmul MCM. The TEM image displayed a spherical shape and uniformly distributed NPs. The in vitro release profile showed low drug release (<7%) in the first 2 h at pH 1.2, while a sustained release was recorded at pH 6.8 with 99.59 ± 5.32% of the drug over 28 h. In an MTT assay, NINTE-NLCs evidenced cytotoxicity and tumor cell growth inhibition whereas in an in vivo animal study they showed enhancing bioavailability via lymphatic uptake.

Recently, researchers investigated different magnetic NPs (MNPs) as drug vectors to improve NINTE solubility, the pharmacokinetic profile and toxicity.

Dhavale et al. [105] prepared magnetic nanoparticles via the co-precipitation method and then 3-aminopropyltriethoxysilane (APTES) was used as a linker for surface functionalization on MNPs at its end for NINTE conjugation carrying this nanocarrier (MNP-APTES-NINTE) for non-small-cell lung cancer. NPS showed a spherical morphology with an average crystalline particle size for MNPs and MNP-APTES-NINTE of about 14 ± 4.4 nm and 17 ± 3 nm. MNP-APTES-NINTE showed an average size with a hydrodynamic diameter of 73.5 nm, a zeta potential of −19.5 mV, PDI value of 0.445 and good colloidal stability. The drug release from the MNP-APTES-NINTE at pH 5.5 and 7.4 at 37 °C was a sustained release with no initial burst effect. At pH 7.4, 16.22% NINTE release was observed within 6 h, while 31.81% of the drug was released at the end of 48 h, and the drug release behavior at pH 5.5 was higher at about 52.8%, which was observed within 6 h and increased up to 85.51% until 48 h. In cell-based assays, NINTE-conjugated MNPs demonstrated dose-dependent antiproliferative activity against non-small-cell lung cancer (NSCLC) A549 cells and no cytotoxicity was observed in healthy HEK-293 cells.

Karade et al. [106] presented a nanoformulation prepared via the surface modification of MNPs through the monolayer coverage of APTES and subsequent loading of NINTE. The MNPs exhibited a pure magnetite (Fe_3_O_4_) phase, a spherical shape and an average size of about 180 nm. The drug loading (DL) was varied according to the ratio of MNPs:NINTE, and it increased linearly as the amount of NINTE increased. For the MNPs:NINTE ratio (1:3), it showed the maximum loading efficiency (79%) and loading capacity (23%) corresponding to the greater conjugation of NINTE. Drug release was carried out at 37 °C under different pH (pH 7.4 and 5.5) levels to mimic physiological and endosomal pH conditions in cancer cells. At pH 7.4, the nanoformulation was quite stable and only a 28% cumulative drug release was observed after 48 h. The low drug release profile was due to the stable imine bond between MNPs-APTES and NINTE. On the contrary, at pH 5.5, the NINTE release was about 50% within the first few hours, and release was up to 85% after 48 h. The nanoformulation exhibited dose-dependent cytotoxicity on the L-132 cell line.

Shukla et al. [107] developed a niosomal formulation of NINTE as inhalable carriers for NSCLC treatment. Niosomes are bilayer spherical drug delivery carriers composed of nonionic surfactants and cholesterol [108]. NINTE-loaded niosomes were prepared using the thin film hydration technique, blending in different ratios of cholesterol:Span60 with or without 1,2-dioleoyl-3-trimethylammonium-propane (DOTAP). Different formulations (labeled F1, F2 and F3) were prepared which contained different ratios of the selected excipients. Their characteristics are given below. For formulation F1 with 9:1 span60: cholesterol ratio, 195.5 ± 5.0 nm. Upon increasing the concentration of cholesterol, the size was found to be reduced with values of 182.4 ± 3.7 nm (for F2, 7:3) and 176.1 ± 4.5 nm (for F3, 5:5). PDI values of all formulations were found to be in the range of 0.02–0.2. The zeta potential of F1 was -60.5 ± 3.6 mV, decreasing span60 concentration in the formulation was reduced the zeta potential to −48.5 ± 5.1 mV (for F2, 7:3) and −40.2 ± 2.5 mV (for F3, 5:5). F1 demonstrated an EE% of 33.4 ± 9.1% and a DL% of 1.5 ± 0.4%, F2 demonstrated an EE% of 73.1 ± 2.7% and DL% of 3.1 ± 0.4%, and F3 demonstrated an EE% of 52.4 ± 4.1% and DL% of 2.4 ± 0.2%. All the formulations showed biphasic drug release behavior and an initial burst release was observed within 30 min followed by a controlled release of NINTE. F1 showed the highest burst release with 60.5 ± 6.9% of the drug released in 30 min followed by slower drug release and F2 resulted in a decreased initial burst release of 50.2 ± 6.6% of the drug, while F3 resulted in further reduction with a 44.4 ± 5.3% initial burst release. The cationically modified niosomes displayed enhanced internalization and pronounced cytotoxic effects against NSCLC cells, resulting in dose reduction for a chemotherapeutic effect, and possessed appropriate aerosolization properties for efficient pulmonary delivery.

## 4. Osimertinib

As previously reported, Osimertinib (OSI) is a third-generation EGFR inhibitor, and it was effective against the T790M resistance mutation with improved blood–brain barrier (BBB) penetration in comparison with first- and second-generation agents such as Afatinib, Gefitinib, Neratinib and Dacomitinib [109]. Furthermore, OSI prevents the development of new brain metastases [110]. OSI-liposome-based delivery can provide a new formulation that can be administered via alternative delivery routes (e.g., intravenous and inhalation).

Skupin-Mrugalska et al. [111] described liposome preparation obtained via thin lipid hydration in which OSI was loaded either passively during hydration (passive loading) or via ammonium sulfate gradient-assisted loading (active loading). The obtained liposomes were constituted by a mixture of egg-PC (l-α Phosphatidylcholine or 1,2-dipalmitoyl-sn-glycero-3-phosphocholine, DPPC or 1,2-distearoyl-sn-glycero-3-phosphocholine, DSPC), cholesterol, and PEG2000-DSPE (1,2-distearoyl-sn-glycero-3-phosphorethanol-amine-N-[methoxy(polyethyleneglycol)-2000] sodium salt) in different molar ratios. DLS studies revealed that liposomes prepared via active loading had diameters in the range of 109.6–125.7 nm, whereas those obtained via passive loading were of a bigger size (114.8 ± 0.8 nm). The PDI for all studied formulations was below 0.1 and the EE% was almost 100% for all formulations. Stability studies at 4 °C in 10 mM PBS (pH 7.4) were conducted and particle size remained constant over a period of 90 days, as assessed via DLS measurements. The determined values of the zeta potential ranged from −10.2 to −11.9 mV and the pH value (7.4 and 6.1) influenced OSI release from liposomes. Interestingly, the time of release of 50% OSI (t50%) decreased at pH 6.1 and the drug release rate was also affected by the character of the prevailing phospholipid. Finally, liposomes composed of egg-PC showed no cytotoxicity and higher antitumor activity against cancer cells with EGFR mutations than free OSI did.

Sawant et al. [112] developed inhalable OSI liposomes formed of DPPC and cholesterol, in which drug loading occurred in the passive and active mode, through the thin film hydration method. The average particle size of OSI passive liposomes and OSI active liposomes was 108.56 ± 16.40 nm and 100.91 ± 8.37 nm, respectively. The PDI was 0.4 and both the formulations showed a zeta potential of +6 mV. The EE% values for active and passive liposomes were 24.92 ± 2.25% and 77.89 ± 3.16%, respectively. At pH 7.4, passive liposomes displayed a 36% initial burst release within one hour, whereas almost 80% was released within 24 h and 94% was released within 48 h. On the contrary, active liposomes showed that 25% of the drug was released in the first 24 h with slow and controlled release observed for 72 h. For stability studies, OSI passive and active liposomes demonstrated about a 1.4-fold increase in particle size at both 4 and 25 °C, revealing that lyophilization would be necessary to extend the shelf life and maintain stability. Nebulized liposomes showed desired aerosol characteristics and presented a possible route for pulmonary delivery. The biological studies showed that the OSI active liposomes prevented colony formation and cell migration, whereas the 3D spheroid assay displayed that this formulation was as effective as free OSI was at tumor suppression after 6 days of treatment.

Using the Box–Behnken design with three independent variables, Gilani et al. [113] optimized OSI nano lipid carriers (OSI-NLCs) that were evaluated for activity against lung cancer. The preparation of OSI-NLCs was carried out using the hot melt emulsification and sonication method. The optimized OSI-NLC (OSI-NLCop) showed a particle size of 162.6 ± 3.5 nm, a PDI of 0.25, a zeta potential of −24.68 mV and an EE% value of 80.2 ± 3.9%. OSI-NLCop showed a biphasic release profile, i.e., an initial fast release and a later sustained release. In particular, the formulation exhibited significantly (*p* < 0.05) high drug release (82.56 ± 5.43%) compared to that of free OSI (24.75 ± 3.59%) after 24 h. In the first 2 h, an initial fast release was observed (23.21 ± 1.98%) due to the presence of OSI on the nanoparticle surface. The stability of OSI-NLCop was evaluated at 4 °C, 25 ± 2 °C/60 ± 5% RH, and 40 ± 2 °C/75 ± 5% RH; the results indicated that no changes in the particle size and EE% at 4 °C, 25 ± 2 °C/ 60 ± 5% RH were observed, whereas at 40 ± 2 °C/75 ± 5% RH highlighted a significant (*p* < 0.05) increase in size and decrease in EE%. Finally, the cell viability study displayed that the prepared NLC system significantly enhanced the effects in terms of reducing the IC_50_ compared to free OSI.

As patients often became resistant to OSI treatment, resulting in cancer recurrence, researchers investigated a combination therapy in which two drugs were used to treat NSCLC and colon tumors [114,115,116].

To overcome OSI-acquired resistance in NSCLC, Chen et al. [114] developed novel NPs loaded with OSI and Selumetinib (SEL). The authors synthetized a PEG-S-SEL conjugate as a prodrug of SEL, composed of a hydrophilic portion (PEG) and hydrophobic portion (SEL) to form micelles for polymer self-assembly. The PEG-S-SEL conjugate represents a SEL prodrug, but also loads and delivers OSI through non-covalent interactions. A film dispersion method was used to prepare OSI + SEL NPs, as well as NPs with a single drug (i.e., OSI NP and SEL NP). The EE% was about 100% for the optimized NP formulations. The particle size for OSI + SEL NP was 43 nm with a PDI of 0.581, that for SEL NP was 77 nm with a PDI of 0.413 and that for OSI NP was 17 nm with a PDI of 0.595. The zeta potential values for these nanocarriers were −30 mV (for OSI + SEL NP), −55 mV (for SEL NP), and −41 mV (for OSI NP), respectively. The efficacy of the NP formulation in a xenograft tumor model established with PC9-AR OSI-resistant NSCLC cells was also evaluated. The nanocarrier studied in this paper was promised to carry out the targeted delivery of OSI and SEL to tumor tissues and thus reduce their toxic side effects.

Wang et al. [115] presented a combination of OSI and Doxorubicin (DOX) as a promising therapeutic strategy for the treatment of NSCLC brain metastases. In this study, the DOX prodrug conjugate was used as the nanocarrier backbone, as it can form, via the organic solvent evaporation method, NPs without any surfactant with high OSI loading. The particle size of nanocarriers was approximately 100 nm with a PDI of about 0.15 and a zeta potential of −40 mV. TEM images showed uniformly spherical morphology and the OSI and DOX loading content reached 28.62% and 34.34%, respectively. The cumulative release of OSI and DOX from NPs reached 63.95% and 62.64% after 8 h, respectively. These results indicated the good stability of the formulation in the circulation system without any premature OSI and DOX release. In addition, the in vitro cellular study indicated that NPs significantly enhanced uptake and penetration across BBB and improved the anti-tumor effect on PC-9 cells. Moreover, in vivo experiments demonstrated that this drug combination nanocarrier significantly prolonged the survival of mice by minimizing the drug’s toxicity to normal cells.

As a preoperative therapy for colon tumors, Chen et al. [116] investigated combined capecitabine (CAP) and OSI therapy, which quickly and efficiently reduced tumor volumes for preoperative chemotherapy. The authors prepared Eudragit S100/CAP-OSI-PLGA microparticles via coaxial electrospraying. The two drugs were inserted into the microparticle PLGA core which was coated with enteric polymer Eudragit. The particle size was around 2 nm and all formulations exhibited a spherical morphology. The DL% and EE% of CAP and OSI were 4.93% and 4.95%, and 92.9% and 93.1%, respectively. The CAP-OSI-PLGA NPs exhibited a sustained drug release of lower than 10% in the first 2 h at pH = 1.0 (gastric simulation), of lower than 50% from 3 to 5 h at pH 6.8 (small intestine simulation) and of up to 80% for a sustained period of 6–48 h at pH 7.4 (colon simulation). After 48 h, 75% drug release was achieved. The treatment with microparticles resulted in fast cellular uptake with strong cellular growth inhibition. In a nude mouse HCT-116 orthotopic colon cancer model, tumor inhibition was 94% within one week, indicating rapid tumor reduction and providing further support for these nanosystems for preoperative chemotherapy.

Chitosan was also considered a polymer for the development of nano drug delivery systems (NDDS) for OSI [117,118].

Hu et al. [117] investigated NPs (OSI-PLGA-COS NPs) constituting PLGA coated with chitooligosaccharides (COS), a polymer obtained from chitosan degradation used to develop positively charged nanoparticles with good biological affinity. The OSI-PLGA NPs were prepared via ultrasonic emulsification and solvent evaporation and then mixed with COS solution, to obtain the desired OSI-PLGA-COS NPs. The average size of OSI-PLGA-COS NPs was 176.6 ± 0.4 nm, which was lower than that of the OSI-PLGA NPs, which was 188.7 ± 1.3 nm. The zeta potential demonstrated that the surface charge of the OSI-PLGA NPs was 1.37 ± 0.53 mV, while after modification with COS, the zeta potential increased significantly to 18.65 ± 0.38 mV. All NPs exhibited nearly spherical core–shell morphologies under a TEM. The nanoparticles were stable at 4 °C and 37 °C in water for 15 days and in serum for 72 h. The EE% values of OSI-PLGA NPs and OSI-PLGA-COS NPs were 87.04 ± 0.73% and 95.22 ± 0.49%, respectively. The cumulative drug release rate was determined at 37 °C in PBS buffer solutions with different pH values (5.0, 6.5 and 7.4), and the release from nanoparticles was sustained under all pH levels in comparison to the profile of the free drug. The nanoparticles displayed a high cellular uptake rate and inhibited cell survival by acting on p-EGFR, PARP, Bak, caspase-9, Bax, and Bcl-2 and promoted apoptosis.

Kumar et al. [118] developed OSI loaded in polycaprolactone (PCL) or chitosan (CHI) NPs to verify their efficacy against NSCLC. The average NP diameter of OSI-PCL and OSI-CHI ranged from 101.3 ± 11.6 to 385.8 ± 26.4 nm, with zeta potential values from −36.4 ± 3.2 to −31.7 ± 3.9 mV, respectively. The percentage yield of OSI-PCL and OSI-CHI was 64.1 ± 6.9% to 89.5 ± 6.7%, the drug content was in the range of 64.08 ± 5.61% to 94.48 ± 6.27%, the EE% was in the range of 94.85 ± 5.39% to 91.25 ± 5.84% and the DL% was in the range of 28.11 ± 2.07% to 34.89 ± 2.86%. The release study was carried out via dialysis in various simulated fluids. In OSI-PCL NPs, drug release started at 4 h at pH 7.4 and reached 80% after 24 h; no drug release was observed at lower pH values. On the contrary, OSI-CHI NPs displayed 85.33 ± 2.91% in vitro drug release at the colon pH of 7.0 after 8 h and 99.98 ± 5.27% release at 24 h. The cellular uptake of OSI-CHI in mutant EGFR-expressing H1975 and PC-9 cells after 4 h was significantly higher compared with to of OSI-PCL. In addition, the OSI-CHI formulation demonstrated enhanced cytotoxicity in the NSCLC cell after 48 h incubation and showed a significant suppression of tumor growth.

A new and innovative pulmonary therapy using a nanoscale-based OSI delivery system was presented by Yang et al. [119]. The authors investigated a formulation constituted by a lipid core (made up of lecithin, cholesterol, 1,2-dipalmitoyl-snglycero-3-phosphoethanolamine-N-(lissaminerhodamine B sulfonyl), and dipalmitoyl-sn-glycero-3-phosphoethanolamine-N-(6-((folate)-amino) hexanoyl) coated with perfluoro-15-crown-5-ether (PFCE), containing OSI (OSI-PFCE-NPs). These NPs when combined with low-intensity focused ultrasound (LIFU) would rapidly diffuse in the lung and gradually accumulate at the tumor site. OSI-PFCE-NPs showed an average hydrodynamic size of 73.9 ± 1.9 nm, a zeta potential of 46.7 ± 1.3 mV and good stability for 8 weeks at 4, 25, or 37 °C. Encapsulation efficiency was 87.38% and OSI release from NPs occurred relatively faster at pH 5.0, to compared to that at pH 6.5 and pH 7.4; in addition, the cumulative drug release rate rapidly increased after LIFU irradiation. In H1975 cells, LIFU irradiation also enhanced OSI cellular uptake and tumor penetration. Cell viability was not affected by PFCE NPs, suggesting that these NPs were suitable for in vivo applications. Finally, in in vivo experiments, OSI-PFCE NPs accumulated in the tumor at a rate 1.8 times higher (*p* < 0.01) than that of the free drug, leading to LIFU irradiation and a further increase in this value.

## 5. Panobinostat

Panobinostat (PANO) is characterized by low oral bioavailability; therefore, intravenous (i.v.) and intraperitoneal administration was used in tumor-bearing mice and rats. The PANO oral bioavailability in dogs was higher than that in rats (6% vs. 33–50%, respectively) and was comparable to that in humans (49%) (Novartis Pharmaceuticals data). PANO has entered in clinical use for the treatment of hematologic and solid tumors, despite its relevant cytotoxicity [120]. Liposomes represent the most frequently studied innovative PANO formulations studied in recent years [121,122,123].

Jose et al. [121] described immunoliposomes (ImmuLipCP) conjugated with the anti-GD2 antibody, a disialoganglioside expressed in human neuroblastoma and loaded with a topoisomerase I inhibitor (7-ethyl-10-[4-(1-piperidino)-1-piperidino]-carbonyloxy-camptothecin, named CPT-11) and PANO. The researchers prepared ImmuLipCP using the thin film hydration method, followed by drug loading throughout repeated freeze/thaw cycles. The particle size of the prepared liposomes was below 200 nm, the polydispersity index (PDI) was about 0.20 and zeta potentials were between 8.3 and 14.7 mV. The morphology of liposomes was spherical in shape with an aqueous core enclosed by a lipid bilayer. The EE% was 57.8 ± 7.2% for CPT-11 and 63.7 ± 12.3% for PANO. The release profiles of CPT-11 and PANO from ImmuLipCP were evaluated in PBS (pH 7.4) at 37 °C; both drugs exhibited a biphasic drug release profile with an initial burst release lasting up to 5 h (PANO was released faster than CPT-11) and a release of 70–75% for both drugs after 24 h. The in vitro experiments on U87DR cell lines confirmed the intracellular uptake of ImmuLip, with a 6-fold increase in uptake that was dependent on the conjugated Ab density on the liposome surface. In addition, tumor-bearing mice treated with ImmuLipCP showed a significant reduction in the tumor growth rate that led to a prolonged survival time.

He et al. [122] presented a liposomal co-delivery system constituting lactoferrin (LF) conjugated with liposomes containing PANO and JQ1, an inhibitor of bromodomain and extraterminal protein BRD4, to treat an immune-inactive CT26 colorectal tumor. The LF-Lipo were prepared via the thin film dispersion method leading to spherical particles of around 200 nm in size with a zeta potential value of +13 mV. The liposomes remained stable at 37 °C in PBS containing 10% fetal bovine serum (FBS). The EE% was 76.4% for PANO and 88.0% for JQ1, and the DL% was 1.8% and 4.2%, respectively. LF-Lipo had a sustained release profile of 60% for the two drugs released after 24 h. The results showed that the combination therapy of the targeting liposome PANO/JQ1 exhibited a synergistic effect on anti-tumor immunity and efficiently remodeled tumor metabolism. Another interesting finding of this work was the demonstration of the in situ formation of albumin corona, around the liposomes, which enhanced the efficiency of targeted delivery. 

Aguiar et al. [123] identified liposomes to overcome the BBB, Aguiar et al. [123] identified liposomes containing RG3, the most promising single-domain antibody (sdAb), and PANO for the treatment of central nervous system (CNS) diseases such as Alzheimer’s, Parkinson’s disease and brain tumors. The encapsulation of PANO in liposomes was performed using an active loading method; the formulation had a mean size of 110 nm and an EE% of 65 ± 2%. To evaluate the cellular cytotoxicity of the unconjugated and BBB-sdAb-conjugated PANO-loaded liposomes against glioblastoma, a cell viability assay was carried out with the glioblastoma cell line LN229. The results showed that liposomes exhibited potent activity and a dose-dependent inhibitory effect on the proliferation of LN229 cells and no cytotoxic activity was observed for PAN-free RG3 and liposome formulations. Furthermore, the authors validated the BBB targeted liposomes in an in vitro model and observed a significant killing effect against the glioma cell line.

Other researchers focused on PANO encapsulation in exosomes to create a new targeted therapy [124,125]. In detail, very recently Shan et al. [124] investigated a nanocarrier tumor-targeted co-delivery that included PANO and a siRNA sequence for the PPM1D gene mutated in 90% of patients affected by diffuse intrinsic pontine glioma (DIPG). The synthesis of the exosome nanocarrier (cEM@DEP-siRNA) required three steps. In the first step, PANO was loaded onto the core of positively charged nanomicelles (DEP) generated from DSPE-PEG_2000_-PEI_25000_ via the filming rehydration method. In the second step, the PPM1D-siRNA was coated on the surface of DEP via an electrovalent interaction. In the final step, the exosomes (EXO) were used to encapsulate DEP-siRNA via coextrusion using a liposome extruder. The characterization results showed that the mean size of cEM@DEP-siRNA (152 ± 10 nm) was larger than that of EXO (80 ± 2 nm) and DEP-siRNA (40 ± 2 nm, and the zeta potential of exosome nanocarrier was about −20 mV, which is similar to that of EXO. In addition, cEM@DEP-siRNA was stable after 48 h incubation with 10% fetal bovine serum. The PANO release from cEM@DEPsiRNA after 24 h did not exceed 30% at pH 7.4. Finally, through in vivo experiment, cEM@DEP-siRNA achieved good tumor growth inhibition and a prolonged survival time in orthotopic DIPG-bearing mice.

In 2019, Sancho-Albero et al. [125] presented palladium-modified exosomes (Pd-EXO) with cell-specific PANO release at tumors sites. Cell-targeting catalytic devices were generated from cancer-derived exosomes using a methodology based on CO-mediated reduction at a low temperature to generate ultrathin Pd nanosheets directly inside the vesicles. The particle sizes of EXO and Pd-EXO were 146.5 and 155 nm, respectively. Pd-EXO remained relatively stable after 72 h at room temperature, indicating good stability for reproducible in vitro and cell-based studies. The intracellular catalytic properties of Pd-EXO were demonstrated via the in situ activation of PANO in lung cancer A549 cells. Furthermore, Pd-EXO not only displayed the capacity to enter the cancer cells, but also the ability to discriminate between them and other cell types.

To increase PANO water solubility and increase its in vivo efficacy against glioma, Singleton et al. [126] encapsulated PANO in pluronic (P407) nanomicelles. The nanomicelles were prepared via an emulsion-mediated process of organic cosolvent evaporation. After evaporation, the EE% was about 70%, while 30% was present as a solvated molecular species. Nanocarrier particles had an average size of about 25 nm and possessed a neutral surface charge as assessed via zeta potential measurements. The in vivo results showed that PANO, when administered in nanomicelles, was effective at prolonging the survival of glioma-bearing animals without any toxicity to neurons or glia cells.

Chaudhuri et al. [127] presented a new nanocarrier constituted by β-cyclodextrin poly (β-amino ester) (cyclodextrin networks, or CDNs) to increase the loading and delivery of PANO. The maximum PANO loading achieved for CDNs was approximately 5% (~22% encapsulation efficiency), and the drug loading did not increase with the addition of more PANO. The particle size was in the micrometer range and the zeta potential was about 15 mV. The PANO release profile was carried out in PBS at 37 °C and all formulations studied provided effective controlled release, with release times ranging between 5 and 24 days according to the different CDN structures. CDN nanoparticles were internalized and delivered bioactive PANO to GL261 cells. Interestingly, PANO incorporated in CDNs enabled the treatment of orthotopic glioblastoma.

## 6. Alectinib and Anlotinib

Alectinib (ALE), a specific second-generation of NSCLC drug therapy, showed a progression-free survival time of several months in NSLC patients in contrast to that of patients undergoing first-line treatments [128]. Recently, Anlotinib (ANLO) was used as a third-line treatment for NSCLC in China; it was orally administered as anti-angiogenic agent that potently inhibited multiple targets, including VEGFR, PDGFR and the FGR fibroblast growth factor receptor. ANLO combined with immunotherapy may synergistically inhibit tumors and improve patient outcomes [129].

Unfortunately, the oral administration of ALE and ANLO may not be feasible in certain medical situations such as respiratory failure or swallowing difficulties, due to carcinomatous meningitis or mediastinal lymph node involvement. For this reason, new routes of administration, and new drug delivery systems were studied.

Park et al. [130] described a novel suspended self-nanoemulsifying drug delivery system (Su-SNEDDS) developed to increase ALE solubility and dissolution rate. ALE solubility was initially evaluated at different pHs, alone and in the presence of various sodium lauryl sulphate (SLS) concentrations. ALE proved to be poorly soluble (<40 µg/mL) at all pHs tested but in the presence of 10% SLS micelles formed and ALE solubility increased up to 754.31 µg/mL. To select the optimal additive for the development of the Su-SNEDDS, ALE solubility was evaluated using various surfactants and oils and the chosen surfactants were Kolliphor RH40 and Kolliphor HS15, whereas Capmul MCM C8 and Capmul MCM C10 were selected as oils. Nanoemulsions without and with ALE were prepared via simple stirring or ultrasonication and stirring. The obtained SNEDDs showed a good dispersion profile with a particle size lower than 200 nm. The ALE solubility of nanoemulsions obtained via simple stirring was lower than that of the formulation obtained via sonication. Drug loading was increased to achieve 10% ALE in Su-SNEDDS. The dissolution test was performed in a 1% SLS pH 1.2 buffer and showed that the dissolution profile of ALE Su-SNEDDS in the steady state reached over 95% within 30 min.

Sheikhi et al. [131] theoretically studied ALE encapsulation in carbon nanotubes (CNTs) to understand the ability of the nanocarrier to absorb and deliver drugs. The authors investigated the (i) non-bonded interaction of ALE with the CNTs using density functional theory (DFT) calculations in the gaseous phase; (ii) the effect of ALE interaction on the electronic properties of CNTs; (iii) NMR analysis and molecular electrostatic potential (MEP) analysis. The results obtained via these calculations confirmed the possibility of non-bonded interactions between ALE and CNTs, thus indicating CNTs as a valuable system with which to deliver ALE to cancer cells.

Nanodelivery systems were studied to reduce ANLO toxicity and side effects and to improve its bioavailability [132,133].

Zhang et al. [132] developed an ANLO-loaded reduction-sensitive nanomicelle with the cyclic RGD peptide (cRGD-ANLO-RM) for the treatment of melanoma and lung metastases. These micelles were prepared via a thin film dispersion self-assembly method. They presented a particle size of about 30 nm with a slightly negative zeta potential of −15.6 mV; TEM analysis revealed a regular spherical shape and uniform dimensions. The EE% was 98.64% and drug loading was 8.98%. The ANLO release rate increased rapidly, and then slowed down; in particular, the free drug was released in 24 h, the cRGD-ANLO-RM released only 50% of ANLO in 24 h. In vitro cell uptake and in vivo biodistribution experiments highlighted that the nanomicelles entered the target site in an accurate, fast, and effective manner, and increased the drug content at the target site through controlled release. In vitro studies also showed that the nanocarrier significantly inhibited melanoma cell proliferation by blocking the VEGF/VEGFR-2 pathway. In in vivo, pharmacodynamics and antipulmonary metastasis studies, the nanomicelles had better efficacy than did ANLO alone.

Gao et al. [133] studied a new hyaluronic acid (HA) hydrogel conjugated with tyramine (Tyr) and ANLO as a novel delivery system that was able to reduce toxicity and improve antitumor activity in a mouse model of Lewis lung cancer (LLC). The synthetized HA–Tyr hydrogel was freeze-dried and dissolved in distilled water (1.0 wt.%) to obtain a colorless, transparent fluid. The ANLO–HA–Tyr hydrogel was formed via the oxidative coupling of Tyr moieties catalyzed by H_2_O_2_ and horseradish peroxidase (HRP). In vitro release studies showed that ANLO was released from the ANLO–HA–Tyr hydrogel by the lysozyme over an extended period. Within 8 h, 95.7% of the free ANLO in the dialysis bag was released into the dialysate. The ANLO–HA–Tyr release rate was about 80% after 48 h. ANLO uptake was low in solid tumor-bearing mice, whereas it was higher in visceral organs. Compared with the free ANLO, ANLO–HA–Tyr hydrogel reduced toxicity to the lungs, liver, and kidneys of C57BL/6J mice. In addition, ANLO–HA–Tyr also inhibited angiogenesis and apoptosis, showing direct anti-tumor effects. Table 3 shows all the nanotechnological systems presented in this review for NINTE, OSI, PANO, ALE and ANLO.

## 7. Conclusions

Indole-based compounds emerged as highly active derivativities endowed with potent antiproliferative activity against various tumors. In this review, six indole drugs (namely, Sunitinib, Nintedanib, Osimertinib, Panobinostat, Alectinib and Anlotinib) were considered among those approved by the FDA and already present in some clinical trials. The pharmacological properties were described together with their limitations (e.g., solubility issues, unwanted side effects, sub-optimal pharmacokinetic and biodistribution profiles). To overcome these difficulties, the possibility of exploiting nanotechnology was shown. For each drug, the nanotechnological formulations, supplied by the literature of the last ten years, were described. The nanocarriers of all indole drugs revealed the advantages of such delivery, that increased the efficacy of the anticancer agent both in vitro and in vivo.

## Figures and Tables

**Table 1:** Selected indole utilized in cancer therapy, number of completed clinical trials, approval years and treated diseases.

Compound	Number of Completed ^1^ Clinical Trials	Approval Date	Competent Authority	Indication/Disease
Sunitinib	309	2006	FDA	Gastrointestinal stromal tumors and advanced renal cell carcinoma (RCC)
		2011		Rare type of pancreatic cancer
		2017		Adjuvant treatment of adult patients at high risk of recurrent RCC
Nintedanib	87	2014	FDA	Interstitial lung disease (ILD) associated with systemic sclerosis or scleroderma (SSc-ILD)
		2019		Idiopathic pulmonary fibrosis (IPF)
		2020		First treatment for chronic fibrosing ILDs with a progressive phenotype
Osimertinib	35	2015	FDA	EGFR T790M mutation-positive non-small-cell lung cancer (NSCLC)
		2017		EGFR T790M mutation-positive NSCLC, full approval
		2018		First-line treatment for EGFR-mutated NSCLC
		2020		Adjuvant treatment of patients with early stage EGFR-mutated NSCLC
Panobinostat	75	2015	FDA	Multiple myeloma
Alectinib	10	2015	FDA	ALK-positive NSCLC
		2017		First-line treatment for ALK-positive metastatic NSCLC
Anlotinib	29	2018	NMPA	Locally advanced or metastatic n NSCLC patients who have undergone progression or recurrence after ≥2 lines of systemic chemotherapy

^1^ The study has ended normally, and participants are no longer being examined or treated [19]. Studies on healthy volunteers have been excluded from the counting.

**Table 2:** Sunitinib nanotechnological carrier systems.

Nano Delivery System	Authors	Year	Ref
Liposome nanoparticles	Passadouro, M. et al.	2014	[65]
	Hu, J. et al.	2016	[66]
	Yang, X. et al.	2018	[67]
	Jiao, Y. et al.	2022	[68]
	Charkhat Gorgich, E.A et al.	2022	[69]
	Lai, X. et al.	2022	[70]
Chitosan nanoparticles	Joseph, J.J. et al.	2016	[72]
	Saber, M.M. et al.	2017	[73]
	Jafari, H. et al.	2021	[74]
	Alinavaz, S. et al.	2022	[75]
	Karimi, M.H. et al.	2022	[76]
Magnetic nanoparticles	Chen, S. et al.	2017	[78]
	Zhang, Z. et al.	2021	[79]
	Torabi, M. et al.	2023	[80]
Solid lipid nanoparticles	Taymouri, S. et al.	2019	[81]
	Khaledian, S. et al.	2021	[82]
	Ahmed, M.M. et al.	2022	[83]
Micellar nanocomplex	Yongvongsoontorn, N. et al.	2019	[85]
	Zeng, X. et al.	2022	[86]
	He, J. et al.	2019	[87]
	Qin, T. et al.	2020	[88]
	Shih, Y.H. et al.	2022	[89]
	Braatz, D. et al.	2021	[90]

**Table 3:** Summary of nano delivery systems for FDA-approved indole derivatives (NINTE, OSI, PANO, ALE and ANLO).

Drug	Nano Delivery System	Authors	Year	Ref
Nintedanib (NINTE)	Albumin nanoparticles	Xu, Y et al.	2021	[99]
		Zha, Q. et al.	2022	[100]
		Xu, Y. et al.	2021	[101]
	Liposome nanoparticles	Kala, S.G et al.	2022	[102]
		Kallus, S. et al.	2018	[103]
	Nanostructured lipid carriers (NLCs)	Patel, P. et al.	2021	[104]
	Magnetic nanoparticles	Dhavale, R.P. et al.	2023	[105]
		Karade, V.C. et al.	2021	[106]
	Niosomes	K Shukla, S et al.	2022	[107]
Osimertinib (OSI)	Liposome nanoparticles	Skupin-Mrugalska, P. et al.	2020	[111]
		Sawant, S.S. et al.	2021	[112]
	Nanostructured lipid carriers (NLCs)	Gilani, S.J. et al.	2022	[113]
	Conjugates	Chen, W. et al.	2021	[114]
		Wang, X. et al.	2020	[115]
		Chen, R. et al.	2022	[116]
	Polymeric nanoparticles	Hu, X. et al.	2020	[117]
		Kumar, S.K. et al.	2022	[118]
		Yang, J. et al.	2022	[119]
Panobinostat (PANO)	Liposome nanoparticles	Jose, G. et al.	2020	[121]
		He, Y. et al.	2022	[122]
		Aguiar, S.I. et al.	2021	[123]
	Exosomes	Shan, S. et al.	2022	[124]
		Sancho-Albero, M. et al.	2019	[125]
	Nanomicelles	Singleton, W.G. et al.	2017	[126]
	b-cyclodextrin networks	Chaudhuri, S. et al.	2021	[127]
Alectinib (ALE)	Self-nanoemulsifyng drug delivery system (SNEDDS)	Park, E.J. et al.	2022	[130]
	Carbon nanotubes (CNTs)	Sheikhi, M. et al.	2019	[131]
Anlotinib (ANLO)	Nanomicelles	Zhang, Y. et al.	2020	[132]
	Hydrogel	Gao, Q. et al.	2020	[133]

## Data Availability

Not applicable.

## Abbreviations

ALCL	Anaplastic large cell lymphoma
ALE	Alectinib
ALK	Anaplastic lymphoma kinase
ANLO	Anlotinib
APTES	3-aminopropyltriethoxysilane
BBB	Blood–brain barrier
BSA	Bovine serum albumin
CDN	Cyclodextrin network
CHI	Chitosan
Chol	Cholesterol
CMC	Carboxymethyl cellulose
CNS	Central nervous system
CNT	Carbon nanotube
COS	Chitooligosaccharides
Cur	Curcumin
DL	Drug loading
DLS	Dynamic light scattering
dPGS-SS-PCL	Dendritic (poly glycerol sulfate)-SS-poly(ε-caprolactone)
dPGS-SS-PLA	Dendritic (poly glycerol sulfate)-SS-poly(lactide)
dPGS-SS-PLGA	Dendritic (poly glycerol sulfate)-SS-poly(lactide-co-glycolide)
DSPC	1,2-distearoyl-sn-glycero-3-phosphocholine
EE	Encapsulation efficiency
EGFR	Epidermal growth factor receptor
EMA	European Medicines Agency
EPOPC	1-palmitoyl-2-oleoyl-sn-glycero-3-ethylphosphocholine
EXO	Exosome
FA	Folic acid
FBS	Fetal bovine serum
FDA	Food and Drug Administration
FGFR	Fibroblast growth factor receptor
FLT3	Fms-like tyrosine kinase 3
FTIR	Fourier transform infrared spectroscopy
HA	Hyaluronic acid
HADAC	Histone deacetylase
HAS	Human serum albumin
HAT	Histone acetylase
HCC	Hepatocellular carcinoma
HPMC	Hydroxypropyl methylcellulose
i.v.	Intravenous
ILD	Interstitial lung disease
IPF	Idiopathic pulmonary fibrosis
LIFU	Low-intensity focused ultrasound
LPHNP	Lipid polymer hybrid nanoparticle
MMSNP	Magnetic mesoporous silica nanoparticles
MNC	Micellar nanocomplex
MNP	Magnetic nanoparticle
mPEG-PCL	Methoxy poly-(ethylene glycol)-poly(ε-caprolactone)
NDDS	Nano drug delivery systems
NINTE	Nintedanib
NIR	Near infrared light
NLC	Nanostructured lipid carrier
NMPA	China National Medical Products Administration
NSCLC	Non-small-cell lung cancer
NsqNSCLC	Non-squamous non-small-cell lung cancer
OSI	Osimertinib
PANO	Panobinostat
PAsp(DBP)	Poly(aspartic acid-dibutyl-1,3-propanediamine)
PBS	Phosphate-buffered saline
PCL	Polycaprolactone
Pd-Exo	Palladium modified exosomes
PDAC	Pancreatic ductal adenocarcinoma
PDGFR	Platelet-derived growth factor receptor
PDI	Polydispersity index
PEG	Polyethylene glycol
PEGEGCG	Poly(ethylene glycol)-conjugated epigallocatechin-3-O-gallate
PFCE	Perfluoro-15-crown-5-ether
PK	Pharmacokinetics
PLA	Polylactic acid
PLGA	Poly(lactic-co-glycolic acid)
RCC	Renal cell carcinoma
RTK	Receptor tyrosine kinases
SEM	Scanning electron microscopy
SLN	Solid lipid nanocarriers
SMA	Styrene-co-maleic anhydride
SPIO	Supermagnetic iron oxide nanoparticles
SSc-ILD	Systemic sclerosis or scleroderma
SNEDDS	Self-nanoemulsifying drug delivery system
SUNI	Sunitinib
TEM	Transmission electron microscopes
TK	Tyrosine kinase
TKI	Tyrosine kinase inhibitor
TPGS	D-alpha-tocopheryl polyethylene glycol 1000 succinate
US	Ultrasound
Vd	Distribution volume
VEGFR	Vascular endothelial growth factor receptors
XRD	X-ray diffraction
